# Functional Recovery After Severe Postoperative Decline in Glioblastoma: The Importance of Reassessing Treatment Eligibility

**DOI:** 10.7759/cureus.117211

**Published:** 2026-09-30

**Authors:** Alain Roberto Barreiros, Idalberto Luis Fernandez Eng, Jennifer Rodríguez Benítez, Luis A Guzman Blanco, Manuel A Guerra Guerrero, Elizabeth Valdes

**Affiliations:** 1 Family and Community Medicine, Rio Hortega University Hospital, Valladolid, ESP; 2 General Medicine, Independent Research, Boston, USA; 3 Family Medicine, Rio Hortega University Hospital, Valladolid, ESP; 4 Neurological Surgery, Universidad de Ciencias Médicas de Granma, Manzanillo, CUB; 5 Community Medicine, Community Medical Group, Lakeland, USA; 6 Medicine, Georgetown American University, Georgetown, GUY

**Keywords:** frontal lobe syndrome, glioblastoma, hypofractionated radiotherapy, idh-wildtype, karnofsky performance status, postoperative intracranial hemorrhage, pseudoprogression, temozolomide

## Abstract

Glioblastoma is the most common and aggressive primary malignant brain tumor in adults, and those arising in the frontal lobe can present with subtle cognitive and behavioral changes, often delaying diagnosis. We present a case of a 67-year-old male patient who presented with a two-week history of psychomotor slowing and progressive difficulty expressing himself, initially concerning for aphasia. Neurological examination suggested a frontal lobe syndrome rather than a true aphasic disorder. Magnetic resonance imaging (MRI) demonstrated a 4.4×3.2×3.8 cm left frontal mass with peripheral enhancement, central necrosis, and extensive vasogenic edema. He underwent left frontal craniotomy with radiologically complete resection; histopathology confirmed isocitrate dehydrogenase (IDH)-wildtype glioblastoma, central nervous system (CNS) World Health Organization (WHO) grade 4. The patient’s postoperative course was complicated by a new intraparenchymal hematoma and parafalcine subarachnoid hemorrhage. Three weeks after surgery, his Karnofsky performance status (KPS) was 40%, initially raising doubts about his candidacy for chemotherapy. He subsequently improved clinically, allowing initiation of hypofractionated radiotherapy (40 Gy in 15 fractions) with concurrent temozolomide, followed by all six planned cycles of adjuvant temozolomide, complicated by fluctuating thrombocytopenia with one asymptomatic nadir of 37,000/µL. Surveillance MRI approximately nine months after surgery showed no evidence of viable tumor recurrence. This case highlights that a severely reduced early postoperative performance status in glioblastoma does not necessarily preclude completion of standard multimodal therapy, and underscores the importance of reassessing functional status and excluding reversible complications, such as postoperative hemorrhage, before treatment is withheld.

## Introduction

Glioblastoma is the most frequent and aggressive primary malignant tumor of the central nervous system (CNS) in adults, with an average annual incidence of approximately 3.19 cases per 100,000 individuals in the United States and comparable rates reported in Europe. It most commonly affects patients between 55 and 75 years of age, with peak incidence in the sixth decade of life, and despite maximal multimodal therapy, median overall survival remains in the range of 14-18 months [1]. Under the 2021 WHO Classification of Tumors of the CNS (5th edition), the diagnostic term “glioblastoma” is reserved for adult-type diffuse astrocytic gliomas that are isocitrate dehydrogenase (IDH)-wildtype and either demonstrate necrosis or microvascular proliferation, or harbor a defining molecular alteration such as a telomerase reverse transcriptase (TERT) promoter mutation, epidermal growth factor receptor (EGFR) amplification, or combined whole-chromosome 7 gain and whole-chromosome 10 loss (the so-called +7/−10 signature); by definition, this entity is CNS WHO grade 4 [2,3].

The clinical presentation of glioblastoma is highly heterogeneous and depends substantially on tumor location. Tumors involving eloquent cortex often produce readily recognizable focal deficits, but frontal lobe tumors can instead present with insidious changes in cognition, behavior, and language that are easily mistaken for a psychiatric or functional disorder, delaying diagnosis [4]. Psychomotor slowing, blunted affect, and word-finding difficulty without a classical aphasic syndrome are recognized, if underappreciated, hallmarks of frontal lobe dysfunction.

Standard first-line management of glioblastoma typically consists of maximal safe surgical resection followed by concurrent radiotherapy and temozolomide, with subsequent adjuvant temozolomide. This approach was established by the landmark trial by Stupp et al. for the European Organisation for Research and Treatment of Cancer (EORTC) and the National Cancer Institute of Canada Clinical Trials Group (NCIC CTG), which demonstrated an improvement in median overall survival from 12.1 to 14.6 months with the addition of temozolomide to radiotherapy [4]. This regimen, commonly known as the “Stupp protocol,” was primarily evaluated in patients with relatively preserved functional status, making treatment decisions more challenging in older patients and those experiencing significant postoperative functional decline. For selected patients who are older, frail, or unable to tolerate conventional fractionation, hypofractionated radiotherapy, such as 40 Gy delivered in 15 fractions, combined with temozolomide may provide a survival benefit compared with radiotherapy alone while reducing the overall treatment duration and represents an accepted alternative to conventional fractionation [5]. Postoperative functional status, commonly assessed using the Karnofsky performance status (KPS) scale, originally described by Karnofsky and Burchenal [6], is an important factor in determining eligibility for multimodal therapy [7,8]. However, an early postoperative KPS may not fully reflect a patient's subsequent functional recovery, and serial assessment of KPS and its trajectory may provide additional information when determining treatment candidacy [9].

We report the case of a 67-year-old man, previously fully independent, who presented with an insidious frontal lobe syndrome later attributed to a left frontal IDH-wildtype glioblastoma. His postoperative course was complicated by a delayed intracranial hemorrhage and a profoundly reduced KPS of 40%, which initially raised doubts about his candidacy for chemoradiotherapy. His subsequent functional recovery allowed him to complete hypofractionated radiotherapy with concurrent and adjuvant temozolomide in full, illustrating both the diagnostic challenge posed by subtle frontal symptoms and the importance of reassessing, rather than anchoring treatment decisions to an early postoperative performance status.

## Case presentation

A 67-year-old man with a medical history of hepatic cirrhosis, obstructive sleep apnea, chronic kidney disease (CKD), and vitamin B12 deficiency presented to the emergency department after a two-week history of progressive changes in behavior and daily functioning. Until approximately two weeks before presentation, he continued to attend music classes and exercise regularly. According to his partner, he had become noticeably slower and stiffer in his movements, as though routine activities required deliberate effort. During the days immediately preceding presentation, he also developed increasing difficulty expressing himself. His speech remained coherent but became slow and halting, causing visible anxiety and facial flushing. Before the onset of his current illness, he had enjoyed a good quality of life and was fully independent in all activities of daily living. There was no history of fever, recent head trauma, nausea, or vomiting. He had no known drug allergies.

Neurological examination and initial neuroimaging

On initial examination in the emergency department, the patient was disoriented to time. His speech was clear and coherent but limited to a few words, initially raising concern for motor aphasia. There was no dysarthria. No meningeal signs or sensory deficits were identified. Pupils were isochoric and reactive, and plantar responses were flexor bilaterally.

A same-day neurosurgical evaluation provided a more specific characterization of his symptoms. The patient was conscious and bradypsychic, with fluent language output but markedly increased latency in his responses. He was able to comprehend and follow simple commands and correctly name objects. His language disturbance was therefore considered more consistent with frontal lobe dysfunction and psychomotor slowing than with a primary aphasic disorder.

Brain computed tomography (CT) performed on the day of presentation revealed a heterogeneous left frontal lobe mass measuring approximately 4.0 × 3.7 × 5.0 cm. The lesion showed predominantly peripheral enhancement with central hypodense cystic-necrotic areas and was surrounded by extensive vasogenic edema involving the left frontal white matter. Associated mass effect compressed the frontal horn of the left lateral ventricle, although midline structures remained centered (Figure 1).

**Figure 1:**
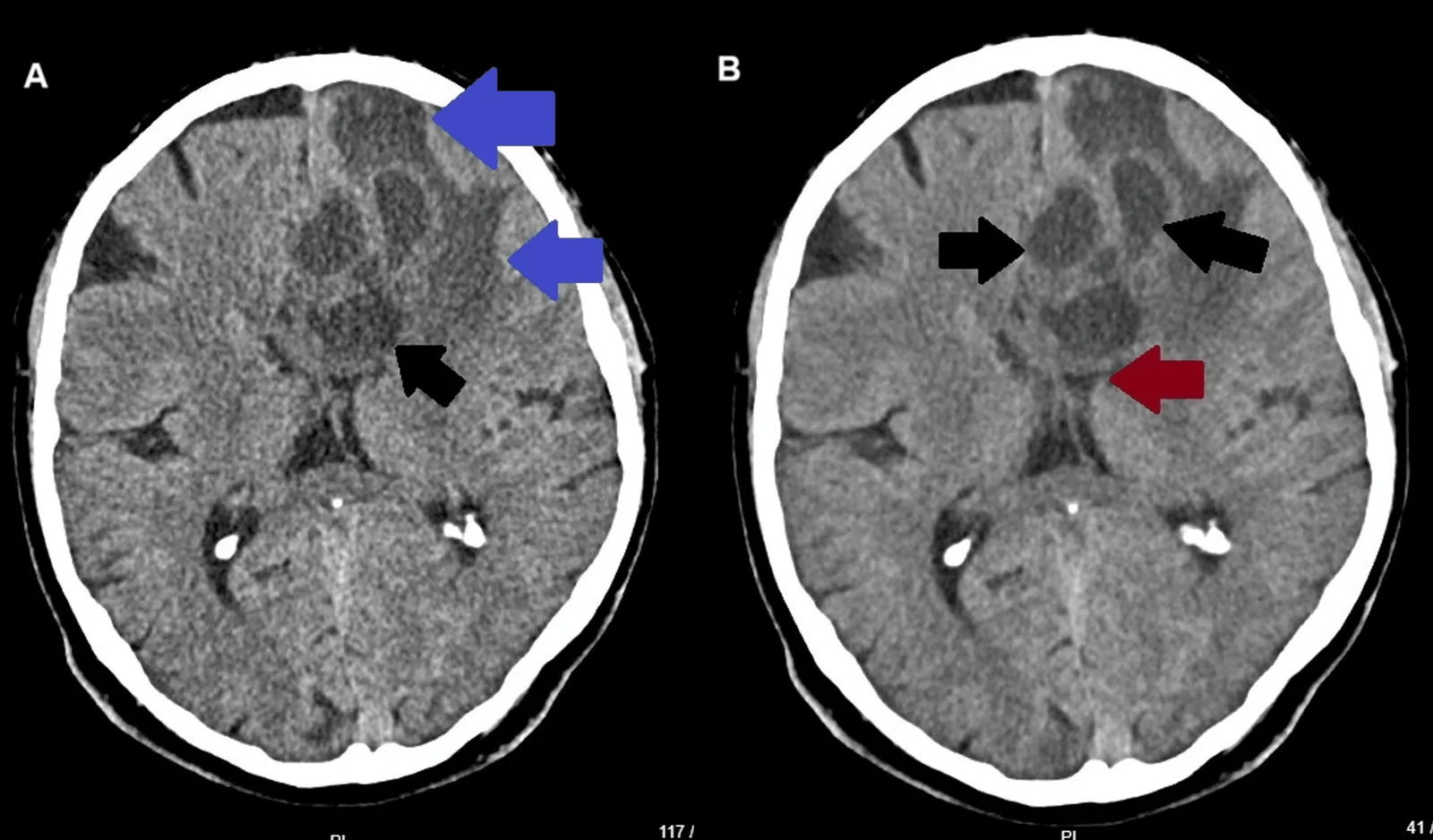
Axial non-contrast head CT images demonstrating a left frontal mass with central hypodense cystic-necrotic areas (black arrows). (A) Extensive surrounding vasogenic edema involving the left frontal white matter (blue arrows); (B) Associated mass effect with compression of the frontal horn of the left lateral ventricle (red arrow), without significant midline shift.

Initial management and surgical treatment

Given the radiological suspicion of a high-grade glial tumor, the patient was started on intravenous dexamethasone to address the significant surrounding edema, receiving an initial 8 mg dose followed by 4 mg every eight hours. Levetiracetam was also initiated for seizure prophylaxis at a dose of 500 mg intravenously every 12 hours. Despite the presence of a relatively large frontal mass with associated mass effect, the patient's clinical condition remained stable. He was conscious, able to follow commands, and had no significant focal motor deficit, signs of raised intracranial pressure, or evidence of acute neurological deterioration requiring emergency surgical intervention.

Following evaluation by the neurosurgical team, the decision was therefore made to proceed with planned elective resection rather than urgent surgery. After overnight observation, during which he remained clinically stable, he was discharged with close neurosurgical follow-up. A dedicated MRI was scheduled for surgical planning and neuronavigation, allowing for a more detailed assessment of the lesion and preparation for resection.

One week later, an MRI performed for surgical planning confirmed an intra-axial parasagittal left frontal tumor measuring 4.4 × 3.2 × 3.8 cm. The lesion was T1-hypointense and heterogeneous on T2 sequences, with peripheral diffusion restriction and prominent intratumoral vascular structures. Following contrast administration, it demonstrated predominantly peripheral enhancement with extensive non-enhancing necrotic areas. Extensive surrounding white matter signal abnormality was also present, consistent with vasogenic edema and/or non-enhancing infiltrative tumor. Mass effect on the left frontal horn persisted without hydrocephalus. The overall radiological impression was that of a high-grade glial tumor (Figure 2).

**Figure 2:**
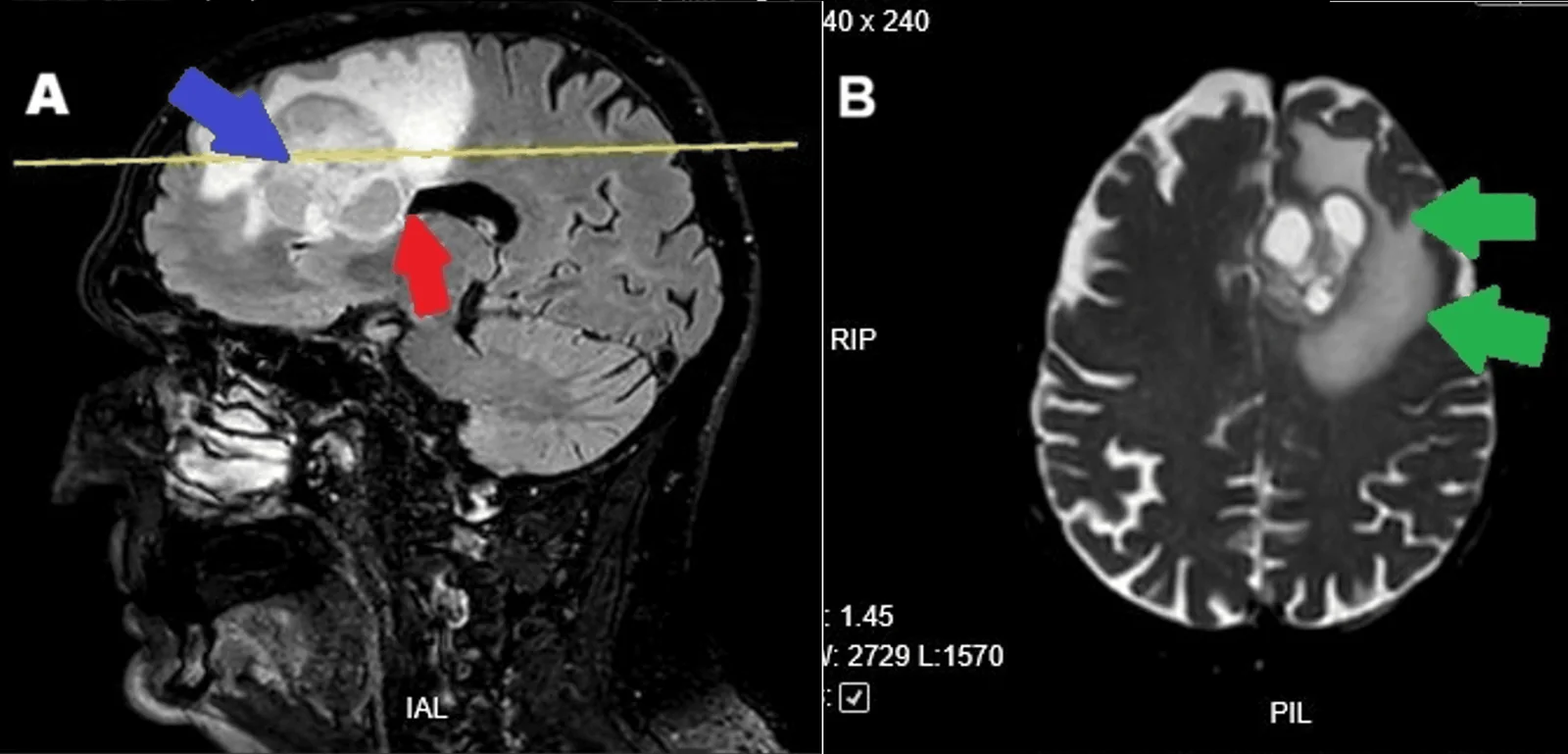
Preoperative brain MRI (A) Left frontal tumor lesion (blue arrow) and compression of the left frontal horn of the lateral ventricle (red arrow); (B) Extensive surrounding vasogenic edema (green arrows).

One day after this MRI, the patient underwent a left frontal craniotomy with neuronavigation-guided tumor resection using an ultrasonic surgical aspirator, with no intraoperative complications reported. An MRI obtained on the first postoperative day demonstrated radiologically complete resection of the contrast-enhancing tumor component. Expected postoperative findings included pneumocephalus, hemosiderin staining, and a 35 × 78-mm extra-axial fluid collection. A persistent non-enhancing perilesional T2/fluid-attenuated inversion recovery (FLAIR) signal abnormality was also noted and scheduled for interval follow-up (Figure 3).

**Figure 3:**
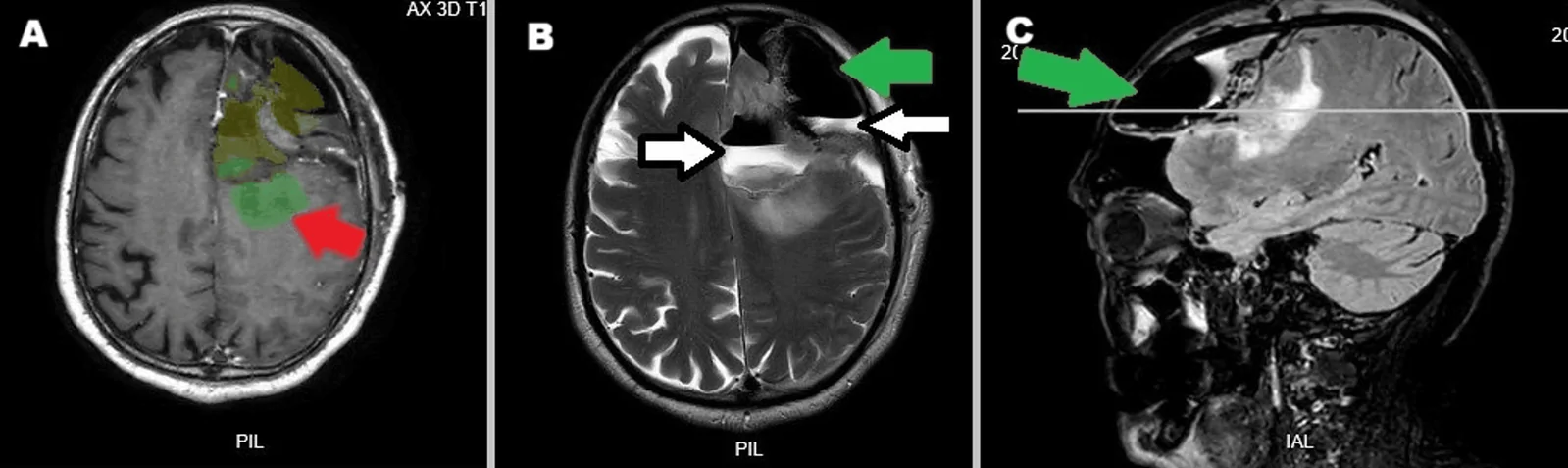
Brain MRI obtained on postoperative day 1 (A) Segmentation overlay corresponding to the residual pericavitary T2/FLAIR signal abnormality (red arrow); (B) Expected pneumocephalus (green arrow) and postoperative extra-axial fluid collections (white arrows with black borders); (C) Expected pneumocephalus (green arrow). FLAIR: fluid-attenuated inversion recovery

Postoperative complication and functional decline

Despite the initially reassuring postoperative MRI, during the following days, the patient developed a marked decline in functional and cognitive status compared with his preoperative baseline. He became less interactive and showed reduced engagement with his surroundings, although he had no focal neurological deficits or signs of meningeal irritation and remained able to follow simple commands. This initial decline was considered compatible with the expected postoperative course and the clinical context of his underlying oncological disease, without features suggesting an acute, severe intracranial complication that would have prompted immediate repeat neuroimaging. Approximately 12 days after surgery, a follow-up CT demonstrated an intraparenchymal hematoma within the surgical bed measuring 2.9 × 2.3 × 2.9 cm (approximately 10 mL), contiguous with a left parafalcine subarachnoid hemorrhage. This was associated with an increase in midline shift from 10 mm to 15 mm and persistent partial effacement of the left ventricular system (Figure 4).

**Figure 4:**
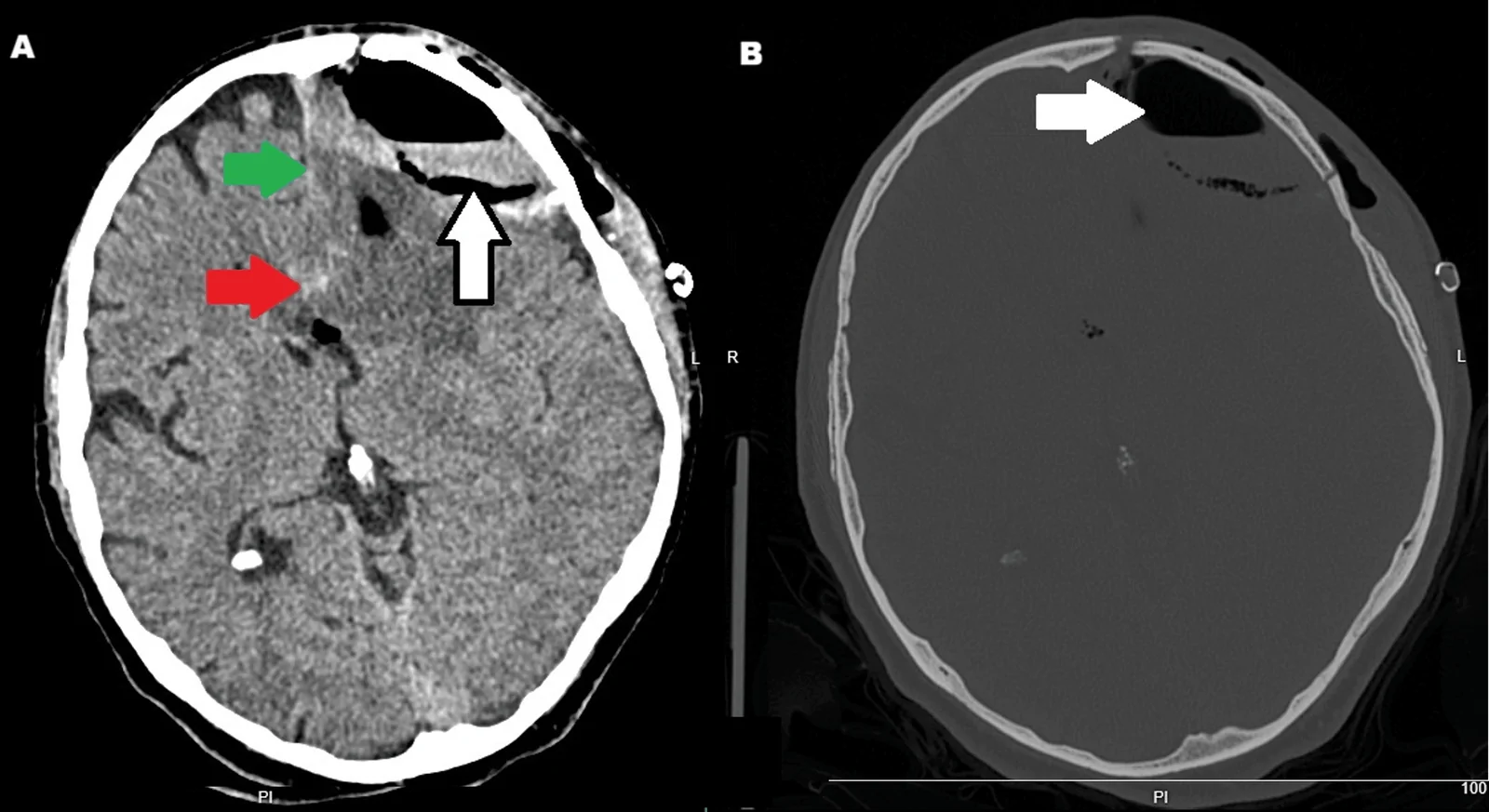
Follow-up head CT approximately 12 days after surgery (A) Increased midline shift (green arrow), contiguous left parafalcine subarachnoid hemorrhage (red arrow), and an intraparenchymal hematoma within the surgical bed (white arrow with black border); (B) Pneumocephalus (white arrow).

Given the relatively limited hematoma volume and the overall clinical context, the multidisciplinary team elected to manage the hemorrhagic complication conservatively with close clinical and radiological observation rather than immediate surgical intervention. The decision reflected the absence of a clear indication for urgent reoperation based solely on the radiographic findings, while also taking into consideration the risks associated with an additional surgical procedure in a patient with significant medical comorbidities. The surgical wound continued to heal appropriately. 

Although the patient did not fully return to his preoperative functional baseline during the hospitalization, his neurological status improved sufficiently to allow discharge. He remained conscious, was able to follow commands, and did not demonstrate signs of acute neurological deterioration or other features requiring continued inpatient management. Dexamethasone and levetiracetam were continued, along with an antiemetic and laxative as needed for supportive care. He was discharged with close follow-up and instructions for continued outpatient reassessment.

At his first oncology evaluation, approximately three weeks after surgery, the patient was wheelchair-bound, although he remained able to walk very short distances independently, described by his family as “step by step.” His relatives considered his condition clearly worse than before surgery, with only minimal improvement during the preceding weeks. On examination, he was conscious but amimic, markedly slowed, partially oriented, and at times appeared disconnected from his surroundings. He was able to follow simple commands and moved all four extremities without an obvious focal motor deficit. His KPS was assessed at 40%.

Given his poor neurological and functional condition at that time, there was understandable concern regarding his ability to tolerate intensive treatment and the potential benefit of chemotherapy. The oncology team discussed these concerns openly with the patient and his family while awaiting definitive histopathological results. At that stage, the patient's condition did not appear favorable for immediate initiation of conventional chemoradiotherapy.

Histopathological and molecular diagnosis

Approximately four weeks after surgery, the final pathological diagnosis was established following completion of the histopathological and immunohistochemical evaluation. Histopathological examination demonstrated a high-grade glial neoplasm characterized by marked cellular atypia, increased mitotic activity, necrosis, and microvascular proliferation, consistent with glioblastoma (Unfortunately, histopathological images were not available for inclusion in this report). The immunohistochemical profile of the tumor specimen is summarized in Table 1.

**Table 1:** Immunohistochemical profile of the resected tumor GFAP: glial fibrillary acidic protein; Olig2: oligodendrocyte transcription factor 2; IDH: isocitrate dehydrogenase; ATRX: alpha-thalassemia/mental retardation syndrome X-linked; p53: tumor protein p53; Ki-67: marker of cellular proliferation

Marker	Result
GFAP	Positive
Olig2	Positive
IDH1	Negative
IDH2	Negative
ATRX	Retained nuclear expression (non-mutated)
p53	Wild-type staining pattern
Ki-67 proliferation index	~20%

Together, these findings established the diagnosis of glioblastoma, IDH-wildtype, CNS WHO grade 4; O6-methylguanine-DNA methyltransferase (MGMT) promoter methylation status was not tested.

Clinical recovery and reassessment of treatment eligibility

While awaiting the final histopathological diagnosis, the patient's condition gradually began to improve. By approximately six weeks after surgery, and two weeks after histopathology had confirmed an IDH-wildtype glioblastoma, he was more consistently oriented and clinically more stable than during his initial oncology evaluation. Although he continued to arrive at clinic in a wheelchair, his family reported meaningful improvement at home, including recovery of his ability to walk.

This clinical evolution prompted reconsideration of the initial treatment approach. His KPS of 40% had reflected his condition during a particularly complicated postoperative period, marked by significant functional decline following surgery and the subsequent hemorrhagic complication. However, his progressive recovery suggested that this early postoperative status might not represent his long-term functional baseline. As his neurological condition improved and the diagnosis of glioblastoma was confirmed, the potential benefits of treatment were reassessed, and the decision was made to proceed with concurrent chemoradiotherapy.

He subsequently received hypofractionated radiotherapy to a total dose of 40 Gy in 15 fractions (2.67 Gy per fraction), together with daily temozolomide at a dose of 140 mg throughout radiotherapy. Dexamethasone and levetiracetam were continued, along with an antiemetic and laxative as needed for supportive care.

The patient tolerated the concurrent phase well. Hematologic and biochemical parameters remained essentially stable, and dexamethasone was successfully discontinued after completion of radiotherapy. Approximately four weeks into treatment, he reported feeling clinically better than before starting therapy.

Treatment-related complications

Four weeks after completing concurrent chemoradiotherapy, the patient transitioned to adjuvant temozolomide at 280 mg/day in two divided doses for five consecutive days of each 28-day cycle. During the adjuvant phase, thrombocytopenia represented the main hematologic challenge. Platelet counts fluctuated throughout treatment, generally remaining below the threshold for dose escalation but without evidence of clinically significant bleeding. Given the persistent thrombocytopenia, the planned dose escalation was not pursued, and the initial temozolomide dose was maintained with close hematologic monitoring (Table 2).

**Table 2:** Concurrent and adjuvant temozolomide course, by cycle

Cycle	Approximate Start	Dose	Platelets at Cycle Assessment	Notes
Concurrent	≈6 weeks after surgery	140 mg/day (with RT)	188,000 → 263,000/µL	Well tolerated; completed 40 Gy/15 fx
1	≈14 weeks after surgery	280 mg/day × 5d	75,000/µL (pre-cycle)	Good hematologic tolerance
2	≈18 weeks after surgery	280 mg/day × 5d	91,000/µL	Grade 1 thrombocytopenia; dose not increased
3	≈22 weeks after surgery	280 mg/day × 5d	100,000/µL	Stable
4	≈26 weeks after surgery	280 mg/day × 5d	125,000/µL (pre-cycle)	Well tolerated
5	≈30 weeks after surgery	280 mg/day × 5d	84,000/µL (pre-cycle); nadir 37,000/µL on incidental ED visit (renal colic, early May)	Transient, asymptomatic, no bleeding; resolved without transfusion
6	≈34 weeks after surgery	280 mg/day × 5d	112,000/µL	Completed; good general condition, no neurological symptoms

Between the fourth and fifth cycles, the patient presented to the emergency department for renal colic. Bloodwork obtained during that visit incidentally revealed an isolated platelet count of 37 × 10⁹/L, representing the lowest documented value during the adjuvant treatment course. He remained clinically asymptomatic, with no evidence of bleeding or other hemorrhagic complications. At subsequent oncology reassessment, his platelet count had recovered.

Given the subsequent recovery and the overall hematologic trend, treatment was continued without omission of any planned cycle, while maintaining the existing temozolomide dose rather than escalating it. No platelet transfusion was required. The patient ultimately completed all six planned cycles of adjuvant temozolomide. By the final cycle, he reported a good general condition, subjective clinical improvement, and no neurological symptoms

Follow-up imaging

Surveillance MRI performed four months after surgery demonstrated expected postoperative changes without evidence of tumor recurrence. The most recent surveillance MRI, nine months after surgery, demonstrated increased T2/FLAIR signal abnormality within the white matter adjacent to the surgical cavity (Figure 5).

**Figure 5:**
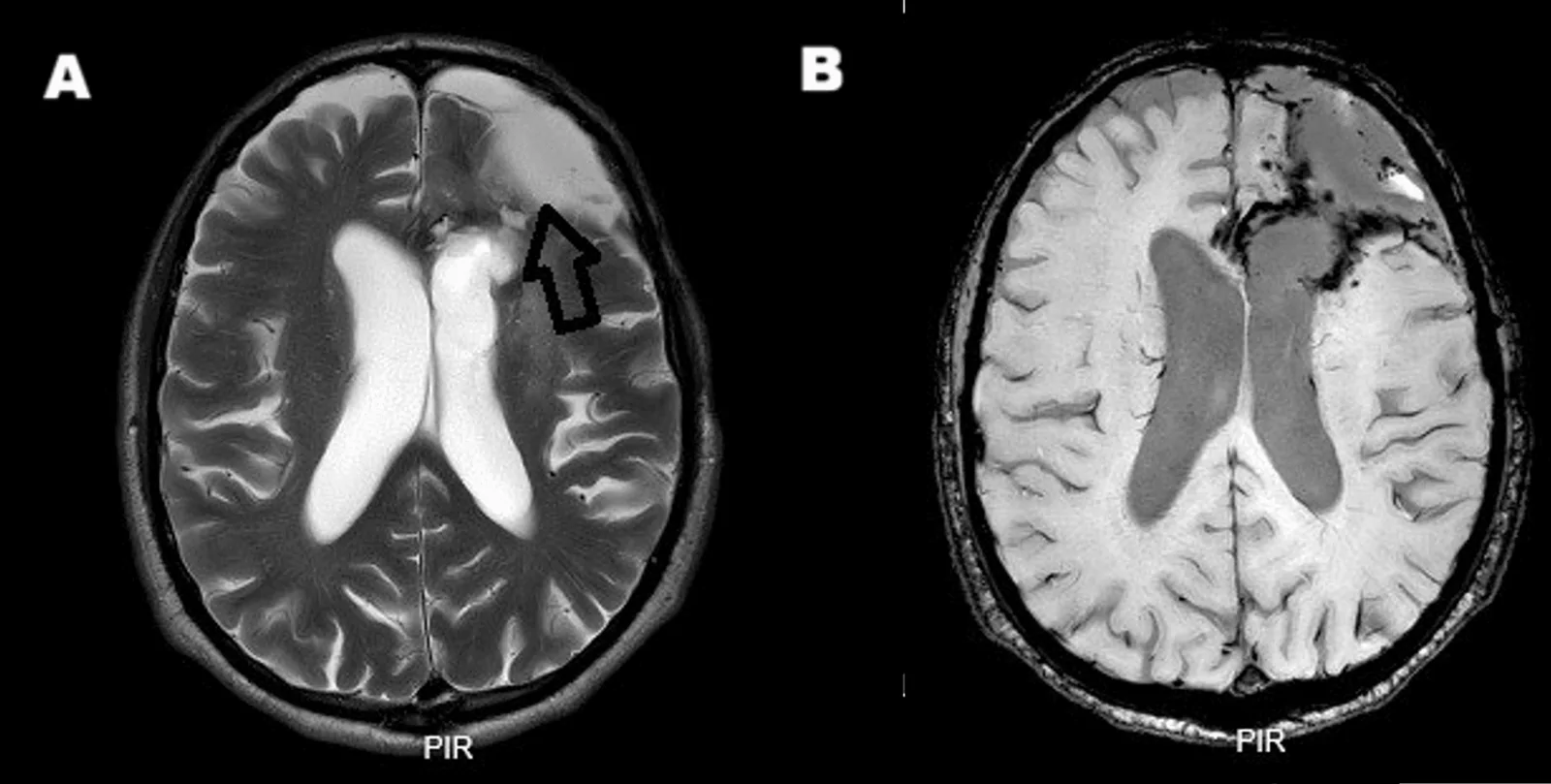
Follow-up brain MRI performed nine months after surgery (A, B) Left frontal postoperative cavity with adjacent T2-hyperintense white matter signal abnormality (arrow in A)

Importantly, these changes were not associated with mass effect, restricted diffusion, contrast enhancement, or an abnormal increase in relative cerebral blood volume on perfusion imaging. Overall, these findings were considered more consistent with treatment-related changes than with tumor progression, with no imaging evidence of residual or viable recurrent tumor.

The timeline of this case from presentation onward is given in Table 3.

**Table 3:** Timeline ED: emergency department; RT: radiotherapy; KPS: Karnofsky Performance Status; SAH: subarachnoid hemorrhage; CNS: central nervous system

Time Point	Event
Two weeks prior to Day 1	Progressive bradypsychia and expressive language difficulty noted by partner
Day 1	ED presentation; CT shows left frontal mass; levetiracetam and dexamethasone started
≈1 week after Day 1	MRI for surgical planning/neuronavigation: findings compatible with high-grade glioma
≈8 days after Day 1	Left frontal craniotomy and tumor resection
≈8 days after Day 1	Immediate postoperative MRI: radiologically complete resection
≈3 weeks after Day 1	Follow-up CT: new postoperative intraparenchymal hematoma and parafalcine SAH, worsening midline shift
≈4 weeks after Day 1	First oncology evaluation: KPS 40%; chemotherapy benefit initially questioned
≈5 weeks after Day 1	Histopathology confirms IDH-wildtype glioblastoma, CNS WHO grade 4
≈7 weeks after Day 1	Clinical improvement noted; starts concurrent hypofractionated RT + temozolomide
≈11 weeks after Day 1	Completes hypofractionated RT (40 Gy/15 fx)
≈15 weeks after Day 1	Six cycles of adjuvant temozolomide (280 mg/day × 5d per 28-day cycle)
≈19 weeks after Day 1	Surveillance MRI: no radiographic findings of tumor progression identified
≈27 weeks after Day 1	Incidental platelet nadir of 37,000/µL during ED visit for renal colic
≈31 weeks after Day 1	Completes sixth and final temozolomide cycle
≈35 weeks after Day 1	Surveillance MRI: signal changes favor treatment effect; no radiographic evidence of viable recurrence

## Discussion

This case illustrates two features that we believe are worth highlighting for clinicians managing similar patients. First, the initial presentation, such as psychomotor slowing and effortful, halting speech in a previously high-functioning man, was easy to misread as an early language or mood disorder rather than a mass lesion. Frontal lobe tumors are known to produce exactly this kind of insidious cognitive and behavioral change, without the more immediately recognizable focal deficits produced by lesions in eloquent cortex, and this can delay recognition [3]. In this patient, the distinction between a frontal syndrome with psychomotor slowing (comprehension, naming, and command-following preserved, with increased response latency) and a true aphasic syndrome, made explicit by the evaluating neurosurgical team, was an important diagnostic clue that prompted timely imaging.

Second, and in our view the more clinically important point, the severity of his early postoperative decline was not fully explained by the resection itself. An immediate postoperative MRI documented a technically complete resection without evidence of restricted diffusion, and it was on follow-up CT roughly 12 days later that a new intraparenchymal hematoma and parafalcine subarachnoid hemorrhage, with progressive midline shift, were identified. Postoperative hemorrhage after cranial tumor resection is not rare: in one recent series of 1,862 consecutive cranial tumor procedures, it occurred in 7.2% of patients, with roughly a quarter of those requiring surgical evacuation, and worse functional outcome scores were associated with its occurrence [9]. In the current case, the hemorrhagic complication was managed medically with close clinical and radiological observation rather than repeat surgical intervention. This approach was considered appropriate in the context of the relatively limited hematoma volume, the absence of an abrupt neurological deterioration requiring emergent evacuation, and the patient's overall clinical and medical risk profile. Importantly, the subsequent clinical course suggests that this postoperative complication likely contributed substantially to his temporary functional decline and KPS of 40%. This case therefore highlights that a severe early postoperative decline in a patient with glioblastoma should prompt careful evaluation for potentially reversible surgical complications before the decline is attributed solely to the tumor or the resection itself and used as a basis for withholding further treatment.

Performance status remains one of the most influential variables in decisions about whether to offer chemoradiotherapy for glioblastoma, and a KPS of 40% at three weeks after surgery is, taken in isolation, a discouraging figure that reasonably prompted our team's initial doubts about the benefit of chemotherapy. However, a recent analysis found that KPS measured later, at hospital discharge, and its degree of improvement over the immediate postoperative course, was more strongly associated with survival than an earlier assessment [8]. Our patient's course is consistent with that observation: his function improved substantially over the following weeks, and it was this trajectory, rather than his nadir KPS, that ultimately determined whether he received treatment. This argues for reassessing functional status dynamically, rather than anchoring a treatment decision to a single early postoperative time point, particularly when a plausibly reversible contributor, such as postoperative hemorrhage, hydrocephalus, or a seizure-related decline, has not yet been excluded or has not yet resolved.

His subsequent treatment, hypofractionated radiotherapy to 40 Gy in 15 fractions with concurrent and adjuvant temozolomide, mirrors the regimen validated by Perry et al. for older or less fit glioblastoma patients considered unsuitable for standard 60-Gy chemoradiotherapy, which improved median overall survival compared with radiotherapy alone (9.3 vs. 7.6 months), with the largest benefit in patients with MGMT-methylated tumors [5]. Compared with the standard EORTC-NCIC regimen [4], this shortened schedule offers a reasonable balance between treatment efficacy and burden for patients whose functional reserve is uncertain, without foreclosing the option of a full six-cycle adjuvant course if they go on to tolerate treatment well, as this patient ultimately did.

Adjuvant temozolomide-associated thrombocytopenia, seen here as a persistent grade 1 abnormality with one asymptomatic grade 3 nadir, is a well-documented toxicity; any-grade thrombocytopenia has been reported in roughly 61% of patients receiving temozolomide for glioblastoma, with severe (grade 3-4) thrombocytopenia in approximately 7-26% [10]. Our patient's course, fluctuating, non-bleeding, and managed with monitoring and dose discipline rather than dose escalation or interruption, illustrates a common and generally manageable pattern that should not, by itself, prevent completion of planned adjuvant cycles.

Finally, the increase in non-enhancing T2/FLAIR signal seen on his most recent surveillance MRI, without mass effect, diffusion restriction, contrast enhancement, or elevated relative cerebral blood volume on perfusion imaging, illustrates the ongoing challenge of distinguishing treatment-related change (including pseudoprogression) from early tumor recurrence in glioblastoma. Perfusion-based metrics such as relative cerebral blood volume are among the most useful adjuncts for this distinction, and their reassuring pattern here supported continued surveillance rather than a change in management [11]. As in most such cases, only close radiologic follow-up, rather than a single time-point assessment, will confirm the benign nature of this signal change.

This report has the limitations inherent to a single retrospective case. Functional status was not formally re-quantified with a KPS score at every subsequent visit, so the qualitative descriptions of clinical improvement, while consistent and corroborated by the patient's family at each encounter, cannot be reduced to a precise numeric trajectory. MGMT promoter methylation status, an important prognostic and predictive biomarker for temozolomide response, was not available in the records reviewed for this report.

## Conclusions

This case highlights that an isolated early postoperative KPS should not be considered the sole determinant of treatment eligibility in patients recovering from major neurosurgical complications. Clinicians should consider structured reassessment of neurological and functional status after an appropriate period of recovery, actively evaluate for potentially reversible causes of decline, and consider the patient's clinical trajectory before making definitive treatment decisions. Further studies are warranted to determine whether standardized longitudinal approaches to performance status assessment could better distinguish transient postoperative impairment from persistent functional limitation and ultimately improve treatment selection decisions in this population.
